# The Diagnosis of an Ovarian Cyst

**Published:** 1901-06-01

**Authors:** 


### Hospital Clinics and Medical Progress.

### THE DIAGNOSIS OF AN OVARIAN CYST.

In a clinical lecture recently delivered at Charing Cross Hospital Dr. Amand Routh[1] directed attention to the various points involved in the diagnosis of an ovarian cyst. The case was a patient with an abdominal tumour, and in such a condition it is useful first of all to get some sort of history. Pregnancy, for example, would have a definite history of its own if it could be relied upon. In the case in question, however, although there was a history of three months' amenorrhœa, the patient was 47 years of age, which rendered pregnancy very unlikely. Moreover, a three months' amenorrhœa does not correspond with such a tumour as was present, rising, as it did, above the navel to the size of an eight months' pregnancy. Again, had the condition been one of ascites, there would have been some history of preceding illness; for whether ascites were due to heart, liver, kidneys, or a local tumour, the patient would be manifestly ill. This patient, however, had not been ill. The use of the catheter excluded the possibility of the case being one of distended bladder, and the amenorrhœa at the age of 47 was against the tumour being a fibroid of the uterus. Returning from this case to the general question, a cyst of this size, reaching a hand's-breadth above the navel, although appearing pretty central, often presents a bulge in one or other direction, as this case did; while such a case seen at an earlier stage, say half or one-third the above size, would occupy a more or less lateral position. In ovarian dropsy, as a rule there is not much bulging of the flanks; but a cyst of considerable size requires a good deal of room, so that the bowels have to be pushed down partly into the flanks, as had happened in this case. In regard to the diagnosis from pregnancy, it was noted that by prolonged watching one could see no alteration of shape, as there would be if it were a pregnant uterus; the intermittent contractions of the pregnant uterus hardening the tumour, so that instead of bulging a little on each side of the spinal column, as it does when it is relaxed, it gets rigid, and is thrown forwards against the abdominal wall.

As against fibroid the signs detected by palpation are of great value. In this case there was no such thing as any very local nodule, although there were large bulges everywhere. Sometimes in fibroids the nodules become sub-peritoneal and a little pedunculated, and thus are quite movable over the surface of the tumour. That is diagnostic of a fibroid tumour of the uterus. In this case there was simply bulging of a uniformly elastic tumour. Palpation also tells whether there are any movements within the tumour such as would be produced by a fœtus.

In regard to consistence most ovarian tumours are uniformly elastic—not hard at one point and soft at another—because their solid portions are, as a rule, on their pelvic aspect. Occasionally, however, one does feel solid lumps in cystic ovarian tumours; and when this occurs, it greatly adds to the difficulty of distinguishing them from fibroids. As to fluctuation, in a unilocular ovarian cyst one can almost always get a wave of fluid the moment one taps it, as is also the case with ascites. But in a multilocular ovarian cyst one may not find it possible to get a distinct wave except over small areas. Thus bulgings, together with localised fluctuations, point to the existence of several large cavities, probably each of them filled with the viscid secretion which is found in adenomatous cysts. Percussion over a cyst, a pregnant uterus, or a fibroid, gives the same dead note, the resonance being in the flanks; whereas in ascites the distribution of the dulness and the resonance is almost exactly reversed. Every now and then, however, these signs mislead us. The colon, for example, may be so loaded and may contain so little gas as to give a dull note down in the flank, or an ovarian tumour may be complicated by the presence of free fluid in the peritoneum; while, on the other hand, even with ascites one may find the flanks resonant from the colon being adherent.

Auscultation showed the tumour in this case to be dumb. The thud of the aorta could be detected, but no sound indicative of pregnancy, and no uterine souffle, such as is often heard in fibroids but very seldom in ovarian tumours. So far the diagnosis from pregnancy and ascites has been chiefly considered, but there are other tumours to be thought of. If a renal tumour, the swelling would be more or less one-sided, would be traceable into the kidney region, and the colon would give a clear note across the tumour. If a splenic tumour, it would be dull to the left ribs, and the tumour would probably be of characteristic shape.

On examining *per vaginam* the uterus appeared a little retroverted and depressed, a position it commonly occupies with ovarian tumours. As an ovarian tumour rises up into the abdomen, it may lie upon an anteverted uterus. As a rule, however, it gets in front of the uterus, which thus is commonly retroverted, with a tendency to lateroversion owing to traction on one corner. Moreover, the uterus can generally by aid of the sound be shown to be about the normal length, to be movable, and to be quite distinct from the tumour. When, as sometimes happens, the uterus is found adherent to the front of the ovarian cyst, and elongated so that the sound may pass 5 inches or more, it is very difficult to be sure that one has not to do with a fibroid tumour in one wall of the uterus, the cavity, with a thinned opposite wall, being in front of it. The distinction may sometimes be made in such cases by the fact that when the uterus is drawn up by the cyst it may be felt lying in front of the cyst above the pubes, and that the sound goes up into it. In other cases one can dip one's hand down, bimanually, and feel the fundus. Such are the main points which make it possible to say that we have to do with an ovarian cyst.

[1] Clin. Journ., May 22.

